# Synchronous Thymoma and Esophageal Cancer Treated With Minimally Invasive Unilateral Video-Assisted Thoracoscopic Surgery: A Case Report

**DOI:** 10.7759/cureus.79020

**Published:** 2025-02-14

**Authors:** Kazuya Okamoto, Shinichiro Kikunaga, Takahiro Karasaki, Yusuke Ogawa, Yu Ohkura, Aya Honda, Sakashi Fujimori, Masaki Ueno, Harushi Udagawa

**Affiliations:** 1 Department of Thoracic Surgery, Respiratory Center, Toranomon Hospital, Tokyo, JPN; 2 Department of Gastroenterological Surgery, Toranomon Hospital, Tokyo, JPN; 3 Department of Gastroenterological Surgery, Toranomon Hospital Kajigaya, Kawasaki, JPN; 4 Department of Research, Okinaka Memorial Institute for Medical Research, Tokyo, JPN

**Keywords:** esophageal cancer, minimally invasive surgery, synchronous neoplasm, thymoma, treatment strategy

## Abstract

The treatment of multi-organ synchronous neoplasms requires a customized strategy for each case. Here, we present our treatment strategy for synchronous double neoplasms involving thymoma and esophageal cancer, which is a rare occurrence in clinical practice. A 68-year-old man was diagnosed with thymoma and advanced esophageal cancer in the middle thoracic esophagus. Following neoadjuvant chemotherapy for esophageal cancer, a concurrent resection of both lesions was performed using minimally invasive unilateral video-assisted thoracoscopic surgery and laparoscopic surgery with gastric conduit reconstruction via the posterior mediastinal route. The patient was discharged on the 14th postoperative day without any adverse events. Minimally invasive, video-assisted unilateral simultaneous surgery for thymoma and esophageal cancer represents a viable therapeutic approach, offering both curative potential and decreased invasiveness. Furthermore, reconstructing the gastric conduit via the posterior mediastinal route was deemed appropriate, as it may help minimize the risk of invasion of the gastric conduit and radiation exposure in the event of thymoma disease progression. Additionally, we propose a treatment strategy flow for synchronous neoplasms located in adjacent multi-organs. This strategy can be applied to various tumor types and may benefit other complex cases.

## Introduction

The treatment of multi-organ synchronous neoplasms requires a tailored strategy for each case. Unlike managing a single lesion, treating multi-organ synchronous lesions involves several key considerations, including indication of perioperative treatment, timing of surgery, and surgical approach [1]. In this case, we discuss a treatment strategy for synchronous thymoma and esophageal cancer.

Esophageal cancer is a challenging malignancy located near critical organs, requiring highly complex surgical procedures and careful perioperative management to avoid complications, which are often associated with a poor prognosis. In contrast, thymoma is a rare malignant neoplasm that originates from the epithelial cells of the thymus in the anterior mediastinum, with an incidence of less than one case per 100,000 individuals [2,3]. Thymoma typically exhibits slow growth and is often detected at an early stage, allowing for curative surgery in many cases, which is associated with a relatively favorable prognosis. Regarding treatment, curative surgery is the standard approach for resectable thymoma, while neoadjuvant chemotherapy followed by surgery is the primary method for resectable esophageal cancer [4,5].

Additionally, the incidence and detection of secondary malignancies in patients with thymoma may be higher than in the general population. The standardized incidence ratio for esophageal cancer is reported as 3.8 (95% confidence interval: 0.5-13.6), indicating a markedly elevated risk compared to the general population [2,6]. Nevertheless, simultaneous occurrences of thymoma and esophageal cancer are exceedingly rare [7]. The synchronous occurrence of both tumors is particularly challenging because they are located in the mediastinum, necessitating careful consideration of perioperative treatment and surgical management strategies.

Here, we report a case of synchronous thymoma and esophageal cancer, successfully treated with complete resection using minimally invasive unilateral thoracoscopic and laparoscopic approaches after neoadjuvant chemotherapy for esophageal cancer.

## Case presentation

**Figure 1:**
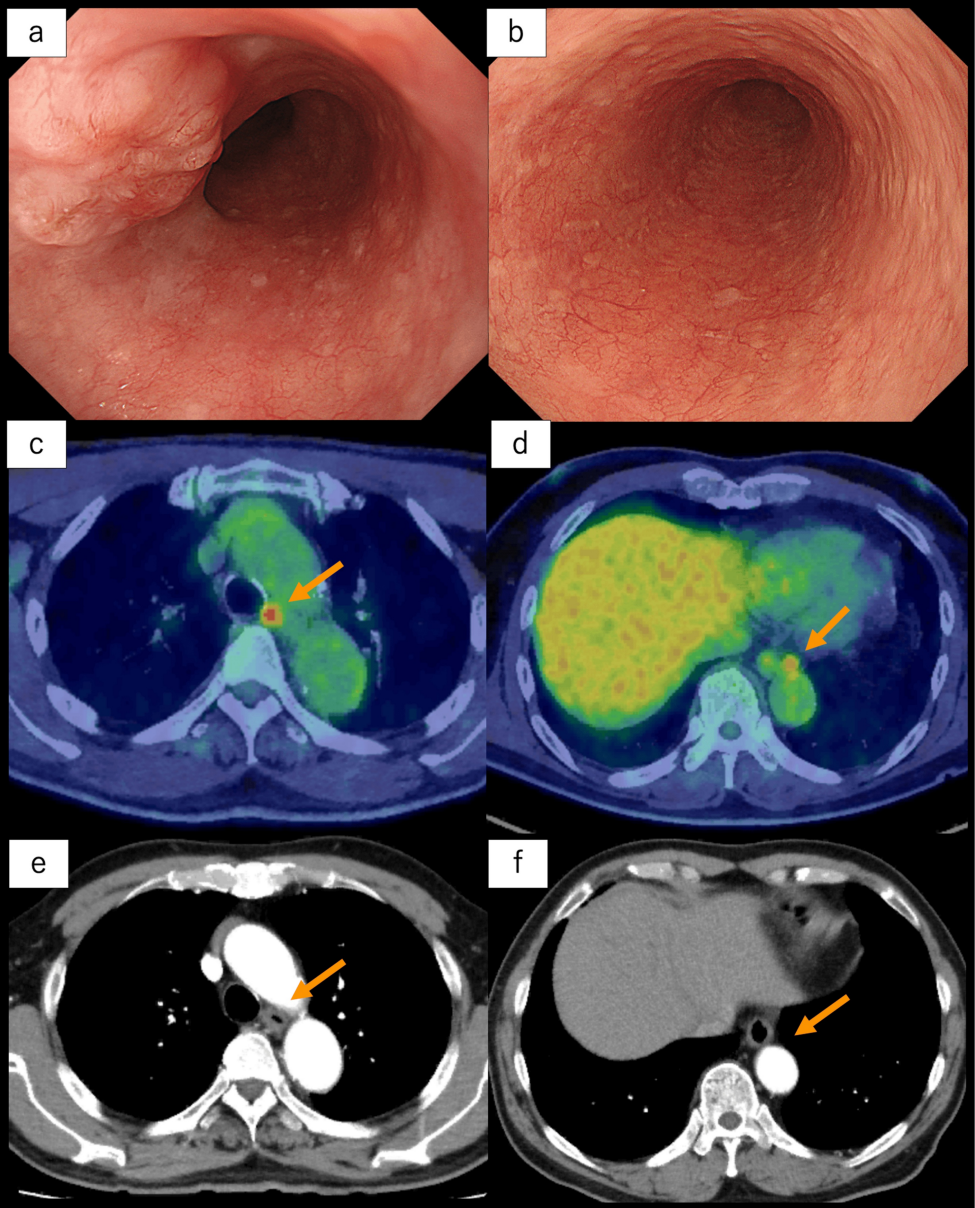
Imaging findings of esophageal primary lesion and metastatic lesions. (a) Endoscopic image showing a 0-IIc lesion, 25 mm in length, in the middle thoracic esophagus, 30 cm from the incisors. A biopsy confirmed the presence of squamous cell carcinoma. (b) Endoscopic image after two courses of neoadjuvant chemotherapy showing complete regression of the esophageal lesion. (c-d) Positron emission tomography-computed tomography scan findings (orange arrows). The images show two lymph node metastases detected at No. 106tbL (SUVmax: 5.43) (c) and No. 112aoA (SUVmax: 4.41) lymph nodes (d). (e-f) A contrast-enhanced computed tomography scan performed after neoadjuvant chemotherapy showed complete remission of enlarged lymph nodes (orange arrows). SUVmax: maximum standardized uptake value.

A 68-year-old man was initially asymptomatic, and esophageal cancer was incidentally detected during an esophagogastroduodenoscopy (EGD) performed as part of a health checkup. A 0-IIc lesion, 25 mm in length, in the middle thoracic esophagus, located 30 cm from the incisors, was observed, and the histological diagnosis was squamous cell carcinoma (Figure 1).

**Figure 2:**
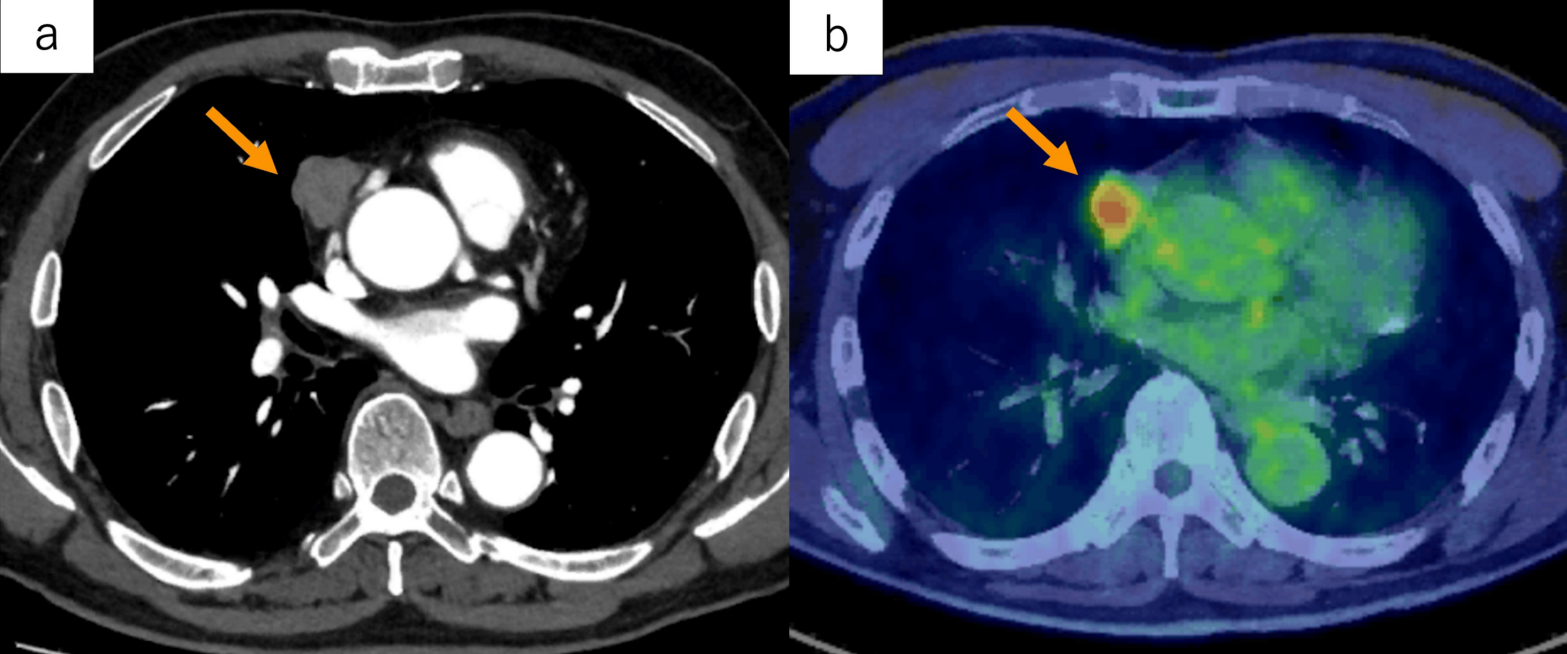
Imaging findings of the anterior mediastinal lesion. (a-b) Computed tomography scan and positron emission tomography-computed tomography scan showing a 27-mm tumor noted at the anterior mediastinum with fluorodeoxyglucose accumulation (SUVmax: 4.65) (orange arrows). SUVmax: maximum standardized uptake value.

Although the esophageal primary lesion was not detected on computed tomography (CT), two enlarged lymph nodes suspicious of metastases were identified at No. 106tbL and No. 112aoA [8]. Additionally, a 27-mm tumor was noted at the anterior mediastinum (Figure 2).

Fluorodeoxyglucose (FDG) positron emission tomography-CT revealed FDG accumulation in the esophageal lesion, enlarged lymph nodes (Figures 1C, 1D), and the anterior mediastinal lesion (Figure 2B). Based on these findings, the patient was diagnosed with advanced esophageal cancer in the middle thoracic esophagus (tumor, node, metastasis (TNM) stage T1bN1M0-II [8]) and an anterior mediastinal tumor suspected to be a thymoma (TNM stage T1N0M0-I [9]). After a multidisciplinary discussion, neoadjuvant chemotherapy for esophageal cancer followed by a one-stage surgical resection of both diseases was planned. As a neoadjuvant treatment for esophageal cancer, a combination regimen of docetaxel, cisplatin, and 5-fluorouracil (DCF: docetaxel 70 mg/m^2^ on day one, cisplatin 70 mg/m^2^ on day one, and 5-fluorouracil 750 mg/m^2^/day on days one to five) was initiated. After two courses of DCF chemotherapy, complete regression of the esophageal lesion was observed in the EGD (Figure 1B), and the CT showed complete remission of enlarged lymph nodes (Figures 1E, 1F). On the other hand, there was no change in the size of the anterior mediastinal tumor after neoadjuvant chemotherapy for esophageal cancer, increasing the likelihood of the anterior mediastinal tumor as a distinct disease from esophageal cancer.

**Figure 3:**
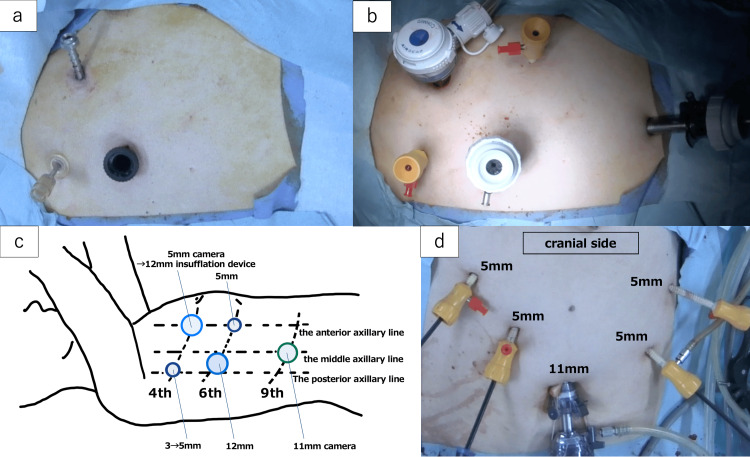
Placement of the ports. (a) Three-port placement during thymectomy; 5 mm, 3 mm, and 12 mm ports were placed at the 4th intercostal space (anterior axillary line), the 4th intercostal space (posterior axillary line), and the 6th intercostal space (posterior axillary line). (b) Five-port placement during esophagectomy; 12 mm, 5 mm, and 12 mm ports were placed at the 4th intercostal space (anterior axillary line), the 4th intercostal space (posterior axillary line), and the 6th intercostal space (posterior axillary line), which were the same port sites used for thymectomy. Additionally, 5 mm and 11 mm ports were placed at the 6th intercostal space (anterior axillary line) and the 9th intercostal space (middle axillary line). (c) Schematic diagram of the port placement (original image created by the author). (d) Five-port placement during laparoscopic abdominal lymphadenectomy and preparation of the gastric conduit.

As planned, the surgery was performed. Under general anesthesia, the patient was placed in the left lateral position. We first performed a right-sided video-assisted thoracoscopic surgery (VATS) thymectomy with three ports (Figure 3A). The port placements were decided to allow the same location to be used for esophageal surgery (Figures 3B, 3C).

**Figure 4:**
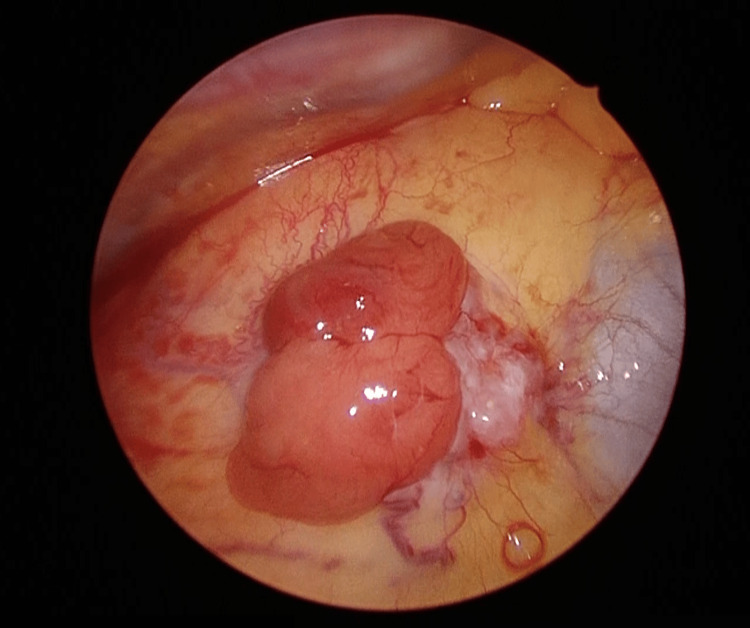
Intraoperative photograph of the anterior mediastinal tumor. The anterior mediastinal tumor had macroscopically extended beyond the mediastinal pleura.

Intraoperatively, there were no adhesions, dissemination, or pleural effusion within the right thoracic cavity. Pleural lavage cytology was not performed. The anterior mediastinal tumor had extended beyond the mediastinal pleura; however, there was no invasion into the lung, phrenic nerve, or pericardium (Figure 4).

The frozen section diagnosis after thymomectomy (i.e., simple removal of the thymoma) was thymoma, and a total thymectomy (i.e., resection of the thymoma and the entire thymus gland) was performed. Subsequently, after two additional ports were inserted, esophagectomy with mediastinal lymphadenectomy was performed via a five-port VATS approach. The abdominal lymphadenectomy and preparation of the gastric conduit were performed laparoscopically (Figure 3D). The intraoperative frozen section analysis showed no evidence of metastasis to the bilateral recurrent nerve lymph nodes, and dissection of the cervical lymph nodes was omitted. The left posterior mediastinal pleura was opened during the esophagectomy. The gastric conduit was reconstructed through the posterior mediastinal route. Two drainage tubes were placed in the right thoracic cavity from the fifth intercostal space along the midaxillary line and in the neck around the cervical anastomosis. The total operative time was 653 minutes, including 70 minutes for the thymectomy, and the total blood loss was 237 ml. No postoperative complications were observed. The patient began oral intake on the 7th postoperative day and was discharged on the 14th postoperative day. Histopathological analysis of the anterior mediastinal tumor confirmed a diagnosis of thymoma type A according to the World Health Organization pathological classification [10], at TNM stage T1bN0M0-I [9] (stage II according to the Masaoka classification [11] and stage III according to the Masaoka-Koga classification [12]), with mediastinal pleural invasion. No pathological response to the preoperative chemotherapy for esophageal cancer was observed in the thymoma. Histopathological examination of the esophagus revealed no residual carcinoma and no lymph node metastasis.

## Discussion

Herein, we report a rare case of synchronous double neoplasms: thymoma and esophageal cancer. The main challenge in treating multi-organ synchronous lesions lies in simultaneously managing two distinct malignancies, considering their individual characteristics, treatment needs, and potential interactions. Careful staging, treatment prioritization, and coordinated management are essential for achieving optimal outcomes and minimizing complications. Pre-existing systemic complications, including past surgical history, accessibility to minimally invasive surgical modality, and the invasiveness of each surgery, should also be considered. It is essential to consult with a multi-disciplinary team while reflecting on the patient’s will to establish an optimal treatment strategy for each case.

**Figure 5:**
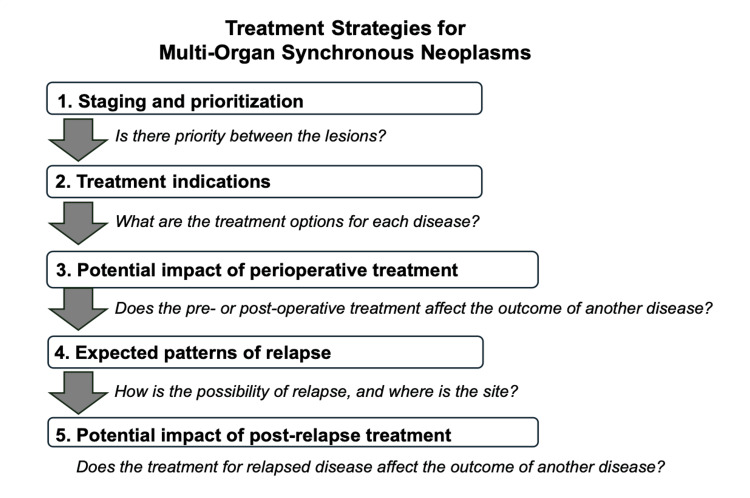
Treatment strategy of synchronous neoplasms located in adjacent multi-organs. A proposed treatment strategic workflow consists of five steps: (1) staging and prioritization; (2) treatment indications; (3) potential impact of perioperative treatment; (4) expected patterns of relapse; and (5) potential impact of post-relapse treatment. Original image by the author.

We propose a treatment strategic workflow for synchronous neoplasms located in adjacent multi-organs consisting of five steps: (1) staging and prioritization; (2) treatment indications; (3) potential impact of perioperative treatment; (4) expected patterns of relapse; and (5) potential impact of post-relapse treatment (Figure 5). This strategic approach applies to various combinations of synchronous neoplasms, including tumors in both adjacent and distant multiple organs, and can be tailored for other complex cases. For the synchronous neoplasms in adjacent organs discussed in the manuscript, concomitant resection from the same incisions or ports may be considered. For the synchronous neoplasms in distant organs, careful consideration should be given to the order and timing of the surgery.

Step 1: Staging and prioritization

The initial diagnosis was advanced esophageal cancer with clinical TNM stage T1bN1M0-II and suspected to be a thymoma with TNM stage T1N0M0-I. The low maximum standardized uptake value (SUVmax) observed in the thymoma indicates a minimally invasive nature of the tumor [13]. In this case, the SUVmax was low for the anterior mediastinal tumor, leading us to suspect that the anterior mediastinal tumor was a thymoma rather than a thymic carcinoma. Furthermore, it was unlikely that the suspected thymoma would become unresectable during the two-month period of neoadjuvant chemotherapy for esophageal cancer. Additionally, monitoring changes from chemotherapy was considered meaningful for treatment planning of the thymoma. Thus, advanced esophageal cancer was considered the prognostic factor, and neoadjuvant chemotherapy for esophageal cancer was prioritized, followed by a one-stage surgical resection of both diseases. Of note, there is a potential variability in the interpretation of imaging study results, especially when the findings are marginal, which may complicate the planning of treatment strategy.

Step 2: Treatment indications

Curative resection of both thymoma and esophageal cancer, if possible, is recommended. In Japan, the standard treatment for resectable advanced esophageal cancer is not definitive chemoradiotherapy (DCRT), but rather neoadjuvant chemotherapy followed by surgery [5]. Furthermore, DCRT treatment may result in adhesions within the thoracic cavity or around the pericardium, increasing the difficulty of thymectomy under a minimally invasive approach.

There are three types in the extent of resection for thymoma: thymomectomy (i.e., simple removal of the thymoma), total thymectomy (i.e., resection of the thymoma and the entire thymus gland), and extended thymectomy (i.e., resection of the entire thymus gland and adjacent mediastinal fat tissue located in front of the pericardium, aorta, and superior vena cava, between the two phrenic nerves, extending from the thyroid gland to the diaphragm, typically applied in cases complicated with myasthenia gravis). There is no consensus on the extent of resection for thymoma without myasthenia gravis yet [2,14]. At our institution, we generally perform total thymectomy for thymoma without myasthenia gravis. Regarding the surgical approach, traditionally, thymectomy has been performed through median sternotomy or lateral thoracotomy. However, recent advancements in minimally invasive techniques, such as VATS and robotic-assisted thoracoscopic surgery, have become increasingly popular, providing better perioperative outcomes, including less blood loss, fewer respiratory complications, and shorter postoperative hospital stays compared to open surgery [15,16]. Consequently, we routinely perform three-port VATS or robotic-assisted surgery for thymoma at our institution. In this case, we successfully performed a one-stage radical resection for both thymoma and esophageal cancer by combining VATS thymectomy with esophagectomy.

Step 3: Potential impact of perioperative treatment

Neoadjuvant chemotherapy with the DCF regimen is considered the standard treatment for resectable locally advanced esophageal cancer [17]. However, recent ongoing trials such as the JCOG1804E and ESCORT-NEO trials have shown that, in the neoadjuvant setting, combination therapy with chemotherapy and immune checkpoint inhibitors (ICIs) may yield superior results compared to chemotherapy alone. These findings suggest that ICI-based regimens may become one of the standard neoadjuvant treatments in the future [18,19]. On the other hand, while ICIs have also demonstrated therapeutic efficacy in thymoma, their use remains controversial due to the high incidence of immune-related adverse events. Due to these concerns, existing reports generally recommend avoiding ICIs in thymoma cases [20,21]. Therefore, it may be advisable to avoid the use of ICIs as part of neoadjuvant chemotherapy in cases of synchronous double lesions involving thymoma and esophageal cancer.

Considering the possibility of postoperative radiation therapy (PORT) to the anterior mediastinum, we selected the posterior mediastinal route for reconstructing the gastric conduit after esophageal resection. The indication for PORT in cases of completely resected thymoma with mediastinal pleural invasion remains controversial [4,22]. Since R0 resection was achieved, PORT was not applied for this case.

Step 4: Expected patterns of relapse & step 5: potential impact of post-relapse treatment

During postoperative follow-up, we must carefully monitor for the recurrence of both thymoma and esophageal cancer. Post-relapse treatments of thymoma and esophageal cancer are summarized in Appendices Table A1. Previous reports suggest that most recurrence cases after esophagectomy occur early, within the first two years [23,24]. However, thymoma is known to be associated with an indolent recurrence pattern, resulting in a longer time to relapse compared to other malignant diseases. The five-year freedom-from-recurrence rate for stage III [12], as in this case, was reported to be 69.4% [25]. In Japan, a follow-up period of at least 10 years is recommended for thymoma [26]. Thymic epithelial tumor recurrence is known to be mostly limited to local and pleural dissemination [27]. This was another reason that the posterior mediastinal route was chosen for reconstructing the gastric conduit. Radiation exposure to the gastric conduit in case of incomplete resection or local relapse should be avoided, and the risk of gastric conduit invasion in the event of local recurrence should be minimized. In this case, the left pleura was opened during the esophagectomy, which may increase the risk of recurrence in the left thoracic cavity. Therefore, careful postoperative follow-up is necessary.

## Conclusions

Treating multi-organ synchronous neoplasms requires a carefully considered strategy, especially when the lesions are located in adjacent organs. Synchronous neoplasms of thymoma and esophageal cancer were successfully treated following the strategic workflow proposed in this manuscript. In the case presented, unilateral VATS for simultaneous thymoma and esophageal cancer lesions may be recommended as a viable approach for achieving curative resection through a minimally invasive technique. Additionally, reconstruction of the gastric conduit using the posterior mediastinal route was deemed appropriate, as it may help minimize the risk of invasion of the gastric conduit and radiation exposure in case of disease progression of thymoma.

## Appendices

Table A1

**Table 1:** Post-relapse treatment of thymoma and esophageal cancer in the current National Comprehensive Cancer Network (NCCN) guidelines.

	NCCN guidelines	Relapse site	Post-relapse treatment
Thymoma	Version 1. 2025	Local recurrence	Surgery and/or systemic therapy and/or radiation
		Pleural metastasis	
		Distant metastasis	Systemic therapy
Esophageal cancer	Version 5. 2024	Locoregional recurrence	Chemoradiation, surgery, systemic therapy
				Distant metastasis	Systemic therapy
